# Spatial clusters of gonorrhoea in England with particular reference to the outcome of partner notification: 2012 and 2013

**DOI:** 10.1371/journal.pone.0195178

**Published:** 2018-04-02

**Authors:** Allen O’Brien, Ellie Sherrard-Smith, Bersabeh Sile, Charlotte Watts, Ian Simms

**Affiliations:** 1 Department of Global Health and Development, London School of Hygiene and Tropical Medicine, London, United Kingdom; 2 HIV and STI Department, National Infection Service, Public Health England, London, United Kingdom; David Geffen School of Medicine at UCLA, UNITED STATES

## Abstract

**Background:**

This study explored spatial-temporal variation in diagnoses of gonorrhoea to identify and quantify endemic areas and clusters in relation to patient characteristics and outcomes of partner notification (PN) across England, UK.

**Methods:**

Endemic areas and clusters were identified using a two-stage analysis with Kulldorff’s scan statistics (SaTScan).

**Results:**

Of 2,571,838 tests, 53,547 diagnoses were gonorrhoea positive (positivity = 2.08%). The proportion of diagnoses in heterosexual males was 1.5 times that in heterosexual females. Among index cases, men who have sex with men (MSM) were 8 times more likely to be diagnosed with gonorrhoea than heterosexual males (*p*<0.0001). After controlling for age, gender, ethnicity and deprivation rank, 4 endemic areas were identified including 11,047 diagnoses, 86% of which occurred in London. 33 clusters included 17,629 diagnoses (34% of total diagnoses in 2012 and 2013) and spanned 21 locations, some of which were dominated by heterosexually acquired infection, whilst others were MSM focused. Of the 53,547 diagnoses, 14.5% (7,775) were the result of PN. The proportion of patients who attended services as a result of PN varied from 0% to 61% within different age, gender and sexual orientation cohorts. A third of tests resulting from PN were positive for gonorrhoea. 25% of Local Authorities (n = 81, 95% CI: 20.2, 29.5) had a higher than expected proportion for female PN diagnoses as compared to 16% for males (n = 52, 95% CI: 12.0, 19.9).

**Conclusions:**

The English gonorrhoea epidemic is characterised by spatial-temporal variation. PN success varied between endemic areas and clusters. Greater emphasis should be placed on the role of PN in the control of gonorrhoea to reduce the risk of onward transmission, re-infection, and complications of infection.

## Introduction

*Neisseria gonorrhoeae*, the causative agent of gonorrhoea, is the second most common bacterial sexually transmitted infection (STI) diagnosed in England. Gonorrhoea diagnoses reached their nadir in the early 1990s (approximately 10,000 in 1993), rose to 41,262 (2015) and subsequently fell to 36,244 in 2016 [1]. The epidemic has been focused on core risk groups including men who have sex with men (MSM), accounting for 49% of diagnoses (17,584) in 2016, and black Caribbeans [2,3]. Infection is also geographically concentrated and strongly associated with deprivation [4,5]. Transmission is perpetuated by higher rates of partner change and complex sexual networks, which can lead to localised outbreaks such as that observed recently amongst young heterosexuals [6,7]. In addition, there has been increasing concern over emerging antimicrobial resistance in gonorrhoea which threatens effective treatment and infection control strategies [8–12].

In England, sexual health services for the confidential diagnosis, treatment and management of STIs are provided free of charge. Partner notification (PN), including provider referral and outcome follow-up, is an essential control strategy as it reduces the risk of onward transmission, re-infection, and complications [13,14]. Since 2012, information concerning PN has been collected through the Genitourinary Medicine Clinic Activity Dataset (GUMCAD) surveillance system. Here we explore the characteristics of gonorrhoea clusters and endemic areas across England using space-time analytical techniques.

## Materials and methods

### Data sources

All commissioned sexual health services are required to report STI tests and diagnoses to Public Health England (PHE) through GUMCAD. The latest iteration of GUMCAD includes reporting through community pharmacies and internet testing; however, when this project was undertaken testing activity and diagnoses made through such services were not captured unless patients were referred to a commissioned or specialist service for ongoing management. Diagnoses of gonorrhoea were extracted from GUMCAD for 2012 and 2013 [15]. This pseudo-anonymised disaggregate dataset included information on patient age, gender, ethnicity, country of birth, sexual orientation, STI testing and diagnoses, HIV status, clinic attended, and attendance date. Diagnoses were coded as either index or partner notified. A positive diagnosis was considered partner notified if the diagnosis occurred 42 days before or after PN, otherwise it was classified as an index case. This timeframe ensured the study captured individuals who may have experienced symptoms after contact with an index case, even if they were tested for other reasons before PN or delayed clinical attendance. A male or female patient positive for gonorrhoea after attending sexual health services due to PN was defined as a male or female PN diagnosis, respectively.

Clinic and patient location were analysed at two geographic area levels: Middle Super Output Area (MSOA; n = 6,791, population range 5,000 to 15,000) and lower tier Local Authority (LA; n = 326, population range 2,224 to 1,074,283) [15–17]. Population denominators for gender, age, and ethnicity available at MSOA and LA levels were used to calculate testing coverage and diagnosis rate [18]. A population denominator for sexual orientation was not available. The Index of Multiple Deprivation (IMD) classification, grouped by quintile, was included [19].

Diagnoses, including repeat infections (restricted to one positive diagnosis every 42 days), were stratified by gender, age, ethnicity, sexual orientation, region of birth, and HIV status. During the study period some individuals transitioned between age groups. Consequently, 2,489,334 subjects were identified across the age groups compared to 2,420,090 unique individuals. To explore gonorrhoea positivity (proportion of tests which tested positive) the dataset was restricted to those attending for gonorrhoea tests.

Analyses were based on de-identified surveillance data held by PHE. In its role providing infectious disease surveillance, PHE has permission to handle data obtained by GUMCAD under Regulation 3 of the Health Service (Control of Patient Information) Regulations 2002. The London School of Hygiene and Tropical Medicine Research Ethics Committee granted ethical approval on June 5^th^ 2014 (Project ID: 7578).

### Statistical analyses

#### Descriptive analyses

The crude diagnosis rate (not controlled for covariates) was calculated for each LA. To be comparable with the demarcations used in PHE STI profiles in previous years, areas with a diagnosis rate 20% above the England average were classified as having a high rate of infection, whereas those 20% below had a low rate [20].

To examine spatial-temporal patterns the data were classified by quarter. The expected quarterly diagnosis rate was derived from the total diagnosis rate for 2012 and 2013 and was assumed to be evenly distributed. Gonorrhoea clusters were investigated by MSOA regions to provide greater detail on spatial-temporal patterns that were otherwise masked at the LA level. The proportion of PN diagnoses in each LA was calculated by comparing the number of PN diagnoses per LA to the national total of all gonorrhoea diagnoses. The observed proportion of PN diagnoses within each LA was then compared to the expected proportion of 0.045% (i.e., the proportion of PN diagnoses if evenly distributed across all LAs).

#### Detection and analysis of endemic areas and clusters

Endemic areas consisted of MSOAs that had a high diagnosis rate for every six month period in 2012 and 2013. To avoid large endemic areas masking small clusters a two-stage procedure was used [21]. After endemic areas had been excluded, potential clusters were identified using a retrospective spatial-temporal SaTScan analysis (S1 Text) [22]. A likelihood ratio test was performed for each cluster, comparing the expected diagnoses from a Poisson distribution to those actually observed inside versus outside the boundary. Potential clusters were detected at the 95% confidence level and a discrete Poisson model was used to adjust for age, gender, ethnicity, and IMD rank. Population weighted centroids for each MSOA were used to identify clusters containing up to 1% of the population instead of the SaTScan 50% default setting [21]. Data for MSOAs contained within a cluster were aggregated. Clusters were then compared in terms of gender, sexual orientation, and PN classification (index or PN diagnosis). Here the locations have been referred to using local place names. When stratified by cluster location, women who have sex with women (WSW) presented low numbers of diagnoses and were grouped with heterosexuals to form a single female category. Data analysis was conducted in SaTScan v9.2 and STATA13 [23,24].

## Results

### Descriptive analysis

Between 2012 and 2013, 2,420,090 patient attendances were recorded in GUMCAD (Table 1). Of the 2,571,838 gonorrhoea tests performed, 53,547 (2.08%, 95% CI: 2.06, 2.10) were positive. A gradual increase in the proportion of positive gonorrhoea diagnoses was observed over 2012 and 2013.The highest number of patients attending GUM services was seen in the 20–34 year age group (62%). Gonorrhoea positivity was 3 times higher among males (3.27%) as compared to females (1.04%), primarily due to higher rates among MSM. Positive gonorrhoea diagnoses in both MSM (11.7%) and HIV positive patients (12.5%) were over 5 times higher than the average for all gonorrhoea tests performed. Although WSW had the fewest gonorrhoea diagnoses (86/5,097 tests), positivity (1.69%, 95% CI: 1.35, 2.08) in this group was slightly higher than heterosexual females (1.04%, 95% CI: 1.02, 1.05).

**Table 1:** Patients attending GUM clinics and diagnoses of gonorrhoea, England: 2012 to 2013.

Variables		Patients attending GUM clinics, n (% of all attendees)	Gonorrhoea tests, n (% of all tests)	Tests positive for gonorrhoea, % (95% CI)
**Gender**	Male	1 123 533 (46.43)	1 199 968 (46.66)	3.27 (3.24, 3.30)
	Female	1 295 991 (53.55)	1 371 545 (53.33)	1.04 (1.03, 1.06)
	Unknown	566 (0.02)	325 (0.01)	1.85 (0.68, 3.97)
**Age (year)**	<13	1 095 (0.04)	271 (0.01)	0.37 (0.01, 2.04)
	13–14	8 416 (0.34)	6 018 (0.23)	1.81 (1.49, 2.18)
	15–19	312 536 (12.56)	325 449 (12.65)	2.24 (2.19, 2.29)
	20–24	686 396 (27.57)	753 726 (29.31)	1.89 (1.86, 1.92)
	25–34	858 611 (34.49)	910 224 (35.39)	2.08 (2.05, 2.11)
	35–44	361 304 (14.51)	346 228 (13.46)	2.39 (2.34, 2.44)
	45–64	239 236 (9.61)	214 147 (8.33)	2.07 (2.01, 2.13)
	>64	20 096 (0.81)	14 374 (0.56)	1.56 (1.36, 1.77)
	Unknown	1 644 (0.07)	1 401 (0.05)	6.35 (5.13, 7.76)
**Sexual orientation**	Heterosexual male	898 037 (37.08)	949 815 (36.93)	1.45 (1.42, 1.47)
	Heterosexual female	1 217 698 (50.28)	1 318 626 (51.27)	1.04 (1.02, 1.05)
	MSM	174 209 (7.19)	209 463 (8.14)	**11.71** (11.57, 11.85)
	WSW	5 785 (0.24)	5 097 (0.20)	1.69 (1.35, 2.08)
	Unknown	126 211 (5.21)	88 837 (3.45)	1.70 (1.61, 1.78)
**Ethnicity**	White	1 798 375 (74.31)	1 901 625 (73.94)	1.99 (1.97, 2.01)
	Black African	109 960 (4.54)	110 117 (4.28)	1.80 (1.72, 1.88)
	Black Caribbean	84 726 (3.50)	114 076 (4.44)	2.97 (2.87, 3.07)
	Black Other	35 435 (1.46)	44 630 (1.74)	2.80 (2.65, 2.96)
	Asian	106 601 (4.40)	108 442 (4.22)	2.09 (2.01, 2.18)
	Mixed	84 497 (3.49)	100 488 (3.91)	2.90 (2.80, 3.01)
	Other	59 272 (2.45)	61 893 (2.41)	2.72 (2.60, 2.86)
	Unknown	141 224(5.84)	130 567 (5.08)	1.71 (1.64, 1.79)
**Birthplace**	UK	1 799 779 (74.37)	1 939 032 (75.39)	1.93 (1.92, 1.95)
	Outside UK	449 945 (18.59)	464 921 (18.08)	2.79 (2.74, 2.84)
	Unknown	170 366 (7.04)	167 885 (6.53)	1.83 (1.76, 1.89)
**HIV status**	Positive	69 892 (2.89)	55 803 (2.17)	**12.53** (12.26, 12.81)
	Negative	2 350 198 (97.11)	2 516 035 (97.83)	1.85 (1.83, 1.87)
**Year & quarter (Q)**	2012 Q1	428 244 (12.02)	308 973 (12.01)	1.90 (1.85, 1.95)
	2012 Q2	415 686 (11.67)	297 937 (11.58)	1.86 (1.81, 1.91)
	2012 Q3	446 778 (12.54)	325 474 (12.66)	2.04 (1.99, 2.09)
	2012 Q4	443 487 (12.45)	320 957 (12.48)	2.15 (2.10, 2.20)
	2013 Q1	443 513 (12.45)	322 633 (12.54)	2.09 (2.04, 2.14)
	2013 Q2	457 569 (12.84)	326 718 (12.70)	2.11 (2.06, 2.16)
	2013 Q3	468 190 (13.14)	339 744 (13.21)	2.18 (2.13, 2.23)
	2013 Q4	459 276 (12.89)	329 402 (12.81)	2.30 (2.25, 2.35)

The crude diagnosis rate was 100.8/100,000 individuals. Highest diagnosis rates were seen in LAs within cities, including London, Manchester, Newcastle-upon-Tyne, Brighton, Bedford, and Leeds (S1 Fig). Diagnosis rates of over 120/100,000 were seen in 43 LAs but the number of LAs with high rates varied by gender: more LAs with high rates were observed among males (n = 61) than females (n = 13) (S2 Fig). LAs with high rates of male diagnoses were also more geographically dispersed.

**Fig 1:**
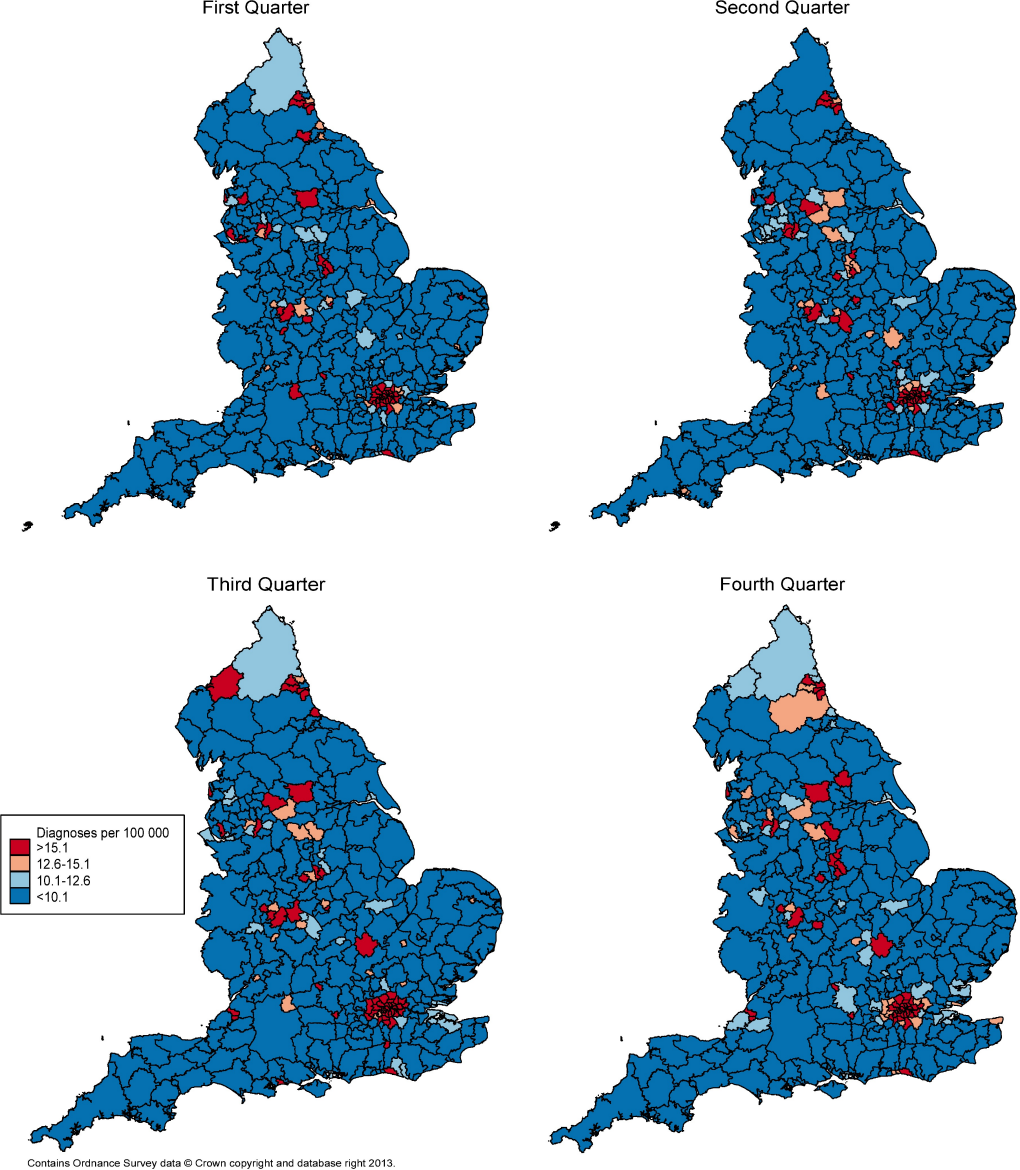
Quarterly gonorrhoea diagnosis rate with regions grouped by Local Authority (LA), England: 2013. Regions are compared to the English average (12.6/100,000 people) as higher, similar or lower. Similar regions were denoted as those within 20% above or below the English average (12.6–15.1 and 10.1–12.6/100,000 people, respectively).

The expected average quarterly diagnosis rate for England was 12.6/100,000 individuals. LAs in London, Brighton, Manchester, and Newcastle-upon-Tyne had persistently high diagnosis rates (>12.6/100,000) across quarters (i.e. three month intervals) whereas diagnoses in surrounding LAs fluctuated (Fig 1). Spatial patterns seen at MSOA level (S3 Fig) generally reflected diagnosis rates seen at LA level, a pattern observed in both 2012 and 2013.

### Partner notification analysis

Of the 53,547 positive gonorrhoea tests, 45,772 (85.5%) were index cases and 14.5% (7,775) were diagnosed as a result of PN. Among index cases, the proportion of gonorrhoea positive males was 3.2 times higher than females (*p*<0.0001). Approximately a third of PN diagnoses were gonorrhoea positive, which is consistent with PHE annual data tables [2]. The observed proportion of PN diagnoses in each LA (calculated from the national total of gonorrhoea diagnoses) was compared to the expected proportion of 0.045% (the proportion of PN diagnoses if distributed evenly across all LAs) (S4 Fig). Female PN diagnoses outweighed male PN diagnoses across LAs; 25% of LAs (n = 81, 95% CI: 20.2, 29.5) had a higher than expected proportion for female PN diagnoses compared to 16% for males (n = 52, 95% CI: 12.0, 19.9).

### Endemic areas and clusters

**Fig 2:**
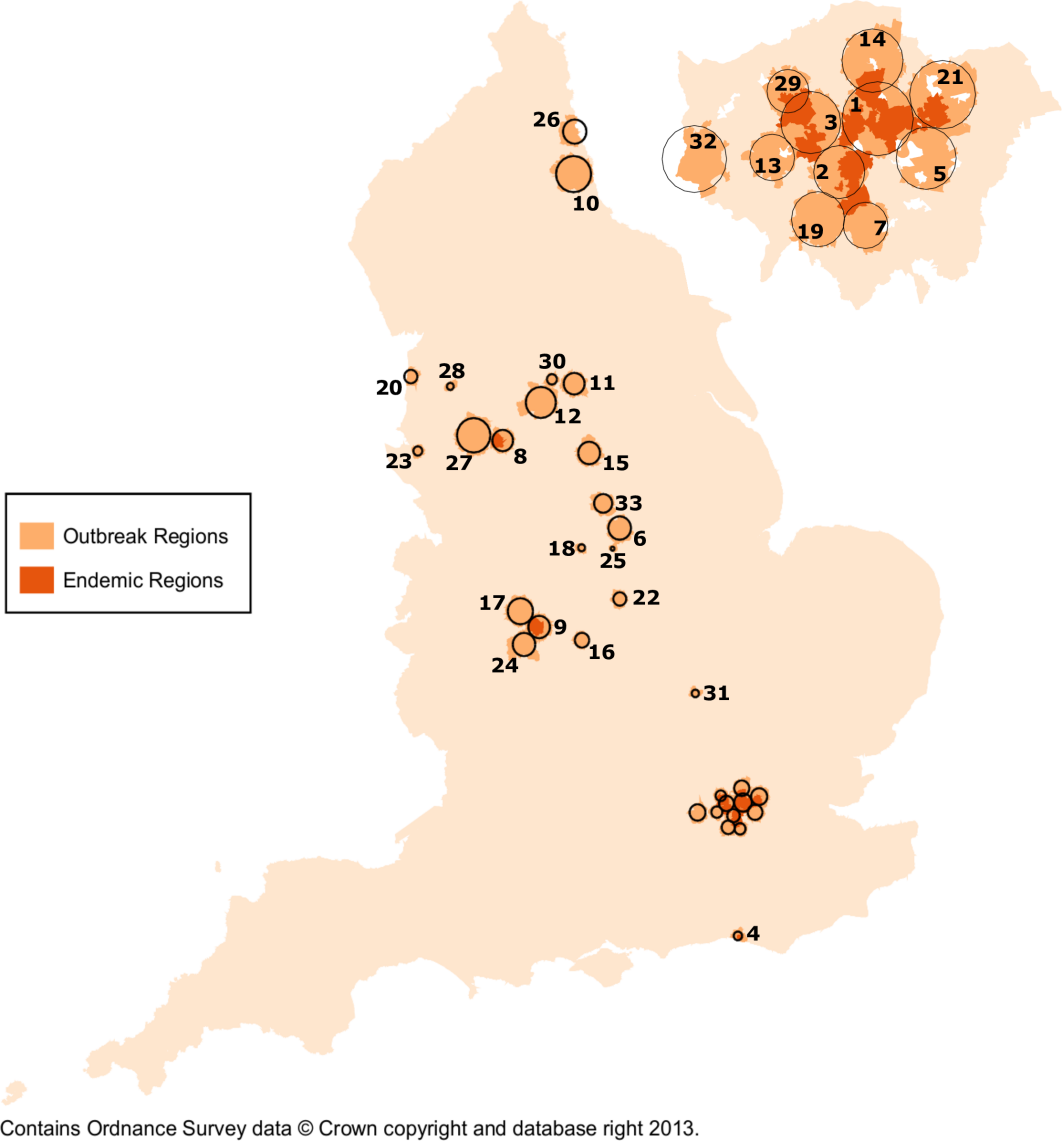
Gonorrhoea endemic regions and outbreak clusters, England: 2012 & 2013. Brighton, London, Birmingham and Manchester were endemic for gonorrhoea by persistently arising in clusters every six months for two years. The London area is enlarged to the top right to better illustrate endemic areas and clusters. Circles denote 33 clusters across 21 aggregate regions (1,137 MSOAs). Numbers next to clusters correspond to region names listed in S1 Table. All outbreak clusters are interpreted as significant at *p*<0.05. Age, gender, ethnicity, and IMD were included covariates.

After controlling for age, gender, ethnicity, and IMD rank, endemic areas were identified in 232 MSOAs (3.7% of England’s population) within 4 cities: London, Brighton, Birmingham and Manchester (Fig 2). These endemic areas included 11,047 gonorrhoea diagnoses (22% of total diagnoses in 2012 and 2013), 86% of which occurred in London (Table 2). A total of 33 clusters were identified across 1,137 MSOAs and spanned 21 towns and cities (approximately 17.2% of England’s population) (S1 Table). Clusters accounted for 17,629 diagnoses (34% of total diagnoses) and ranged from 0.6% (Preston) to 51.9% (London). Although the majority of clusters were adjacent to MSOAs of endemic areas, exceptions were observed around endemic areas in Newcastle-upon-Tyne, Bedford, Leeds, Nottingham, and Derby.

**Table 2:** Characteristics of endemic areas and clusters for gonorrhoea, England: 2012 and 2013.

Region		Summary of gonorrhoea diagnoses				Summary of partner notified gonorrhoea diagnoses*			
		Female diagnoses, n (%)	Heterosexual male diagnoses, n (%)	MSM diagnoses, n (%)	Total diagnoses, n (%)**	Female diagnoses from PN, n (%)	Heterosexual male diagnoses from PN, n (%)	MSM diagnoses from PN, n (%)	Total diagnoses from PN, n (%)**
**Endemic areas**	London	1 214 (12.8)	1 346 (14.2)	6 831 (72.1)	9 477 (100)	175 (14.4)	152 (11.3)	1 213 (17.8)	1 554 (16.4)
	Manchester	89 (12.0)	87 (11.7)	563 (76.1)	740 (100)	16 (18.0)	9 (10.3)	63 (11.2)	88 (11.9)
	Brighton	5 (2.6)	13 (6.7)	174 (90.2)	193 (100)	1 (20.0)	2 (15.4)	24 (13.8)	28 (14.5)
	Birmingham	195 (30.6)	202 (31.7)	167 (26.2)	637 (100)	44 (22.6)	42 (20.8)	37 (22.2)	131 (20.6)
	*Total endemic areas*	1 503 (13.6)	1 648 (14.9)	7 735 (70.0)	11 047(100)	236 (15.7)	205 (12.4)	1 337 (17.3)	1 801 (16.3)
**Clusters**	London	1 847 (20.2)	1 811 (19.8)	5 369 (58.7)	9 147 (100)	255 (13.8)	156 (8.6)	898 (16.7)	1 322 (14.5)
	Manchester	228 (28.9)	198 (25.1)	359 (45.6)	788 (100)	22 (9.6)	19 (9.6)	38 (10.6)	80 (10.2)
	Brighton	29 (5.8)	36 (7.3)	430 (86.7)	496 (100)	6 (20.7)	6 (16.7)	72 (16.7)	84 (16.9)
	Birmingham	463 (41.5)	305 (27.3)	207 (18.5)	1 117 (100)	106 (22.9)	52 (17.0)	48 (23.2)	222 (19.9)
	Bedford	48 (43.6)	41 (37.3)	21 (19.1)	110 (100)	**23 (47.9)**	**25 (61.0)**	8 (38.1)	**56 (50.9)**
	Derby	54 (40.3)	56 (41.8)	21 (15.7)	134 (100)	18 (33.3)	21 (37.5)	**9 (42.9)**	49 (36.6)
	Northumberland	86 (57.0)	55 (36.4)	10 (6.6)	151 (100)	15 (17.4)	17 (30.9)	1 (10.0)	33 (21.9)
	Nottingham	357 (48.4)	290 (39.3)	72 (9.8)	737 (100)	57 (16.0)	24 (8.3)	10 (13.9)	94 (12.8)
	Newcastle-upon-Tyne	365 (43.5)	281 (33.5)	192 (22.9)	839 (100)	54 (14.8)	45 (16.0)	31 (16.1)	130 (15.5)
	Sheffield	175 (46.4)	143 (37.9)	59 (15.6)	377 (100)	34 (19.4)	25 (17.5)	8 (13.6)	67 (17.8)
	Walsall/Sandwell	287 (40.5)	297 (41.9)	112 (15.8)	708 (100)	52 (18.1)	31 (10.4)	21 (18.8)	105 (14.8)
	Coventry	124 (39.5)	118 (37.6)	72 (22.9)	314 (100)	21 (16.9)	12 (10.2)	10 (13.9)	43 (13.7)
	Blackpool	46 (26.3)	49 (28.0)	79 (45.1)	175 (100)	8 (17.4)	4 (8.2)	9 (11.4)	22 (12.6)
	Leicester	141 (40.6)	115 (33.1)	88 (25.4)	347 (100)	16 (11.3)	10 (8.7)	10 (11.4)	36 (10.4)
	Preston	45 (42.5)	37 (35.0)	24 (22.6)	106 (100)	7 (15.6)	9 (24.3)	3 (12.5)	19 (17.9)
	Bradford	36 (31.9)	5 (4.4)	23 (20.4)	113 (100)	8 (22.2)	**0 (0)**	3 (13.0)	14 (12.4)
	Bolton/Wigan/ Warrington	203 (40.4)	170 (33.8)	128 (25.4)	503 (100)	17 (8.4)	16 (9.4)	9 (7.0)	42 (8.3)
	Leeds	259 (38.5)	219 (32.5)	193 (28.7)	673 (100)	20 (7.7)	20 (9.1)	16 (8.3)	56 (8.3)
	Calderdale/Kirklees	218 (47.7)	163 (35.7)	65 (14.2)	457 (100)	19 (8.7)	18 (11.0)	6 (9.2)	45 (9.8)
	Ashfield/Mansfield	54 (47.8)	33 (29.2)	26 (23.0)	113 (100)	1 (1.9)	3 (9.1)	2 (7.7)	6 (5.3)
	Liverpool	74 (33.0)	77 (34.4)	73 (32.6)	224 (100)	**1 (1.4)**	5 (6.5)	**1 (1.4)**	**7 (3.1)**
	*Total cluster areas*	5 139 (29.2)	4 499 (25.5)	7 623 (43.2)	17 629 (100)	760 (14.8)	518 (11.5)	1 213 (15.9)	2 532 (14.3)

*Bold values indicate the highest or lowest percentage of diagnoses that were partner notified.

**Total diagnoses include cases with unknown sexual orientation.

The average age (year) of females, heterosexual males, and MSM was 24.4, 28.4, and 32.7, respectively, across the 4 endemic areas and 23.4, 27.6 and 32.4 across the 33 clusters. The proportion of gonorrhoea diagnoses due to PN was similar between individuals with HIV (15.6%) and without HIV (16.5%). Diagnoses seen in heterosexual males and females were similar across clusters (0.7% difference, *p* = 0.84). Among females, the proportion of PN positive diagnoses ranged from 1.4% (Liverpool) to 47.9% (Bedford) and from 0% (Bradford) to 61% (Bedford) in heterosexual males (Table 2). Heterosexual males accounted for the highest proportion of all diagnoses in Derby and Walsall/Sandwell (both 42%). There was no observable statistical difference or pattern in the proportions of PN diagnoses between heterosexual males and females after controlling for age, gender, ethnicity, and IMD rank. Some areas were dominated by heterosexually acquired infections (Northumberland), others by diagnoses seen in MSM (Brighton). In Brighton and London, diagnoses seen in MSM accounted for over 85% and 65% of all diagnoses, respectively.

## Discussion

Spatial-temporal variation is a critical feature of the gonorrhoea epidemic in England. The majority of gonorrhoea diagnoses were observed in urban areas [25], particularly London, Brighton, Manchester, and Birmingham. Although the endemic areas generally corresponded to locations with higher MSM populations (London, Manchester and Brighton), clusters were relatively common among sexual networks of both MSM and heterosexuals [21]. Both endemic areas and clusters overlapped with some of the most deprived areas of England (Liverpool, Manchester, Blackpool, Birmingham, Bradford and the London Borough of Hackney) [26]. Whilst more males presented as index cases to clinical services, a greater proportion of partner notified attendees were female. The value of PN as a case detection strategy was observed in the high proportion of gonorrhoea cases detected using this method; however, this varied considerably with respect to location, sexual orientation, and gender [13,14].

The MSOA analysis highlighted clusters that would otherwise have remained undetected at the LA level. SaTScan analysis is advantageous since it does not require an *a priori* hypothesis about cluster location, size or duration. The software adjusts for multiple testing and inhomogeneous population density. Additionally, since the technique is not dependent upon administrative unit denominators, the analysis was not influenced by the modifiable areal unit problem [27]. Although GUMCAD captures most gonorrhoea testing and diagnostic activity, including index cases (patients diagnosed first at GUM clinics) and their partners, these cannot be directly linked within the dataset [15,27,28]. Consequently, only the analysis of relative proportions of partner-notified to index based diagnoses can be explored. Whilst this captures current PN practices, interpretation is limited. Furthermore, when the study was conducted only 2012 and 2013 GUMCAD data was available. Although these results accurately describe the recent landscape of gonorrhoea diagnoses and PN across England, they can only approximate the current status due to possible changes in clinic locations, administrative boundaries, and underlying demographics. The new version of GUMCAD (currently under development) will capture more detailed PN information [29]. Recent studies have demonstrated the ability to predict gonorrhoea prevalence, including the development of online tools that predict gonorrhoea prevalence for non-GUM clinics [30,31].

High rates of gonorrhoea are typically distributed within core groups in densely populated urban areas [32]. These endemic areas act as reservoirs that seed clusters into bordering regions. This population structure is characteristic of a meta-population; gonorrhoea endemic populations sustain the epidemic and seed sexual networks in smaller satellite communities through bridging populations [33–35]. Infection cycles are relatively independent but a cluster is more likely to become extinct in smaller populations [36].

Identifying endemic areas and clusters and exploring the characteristics of local epidemics is an essential aspect of developing effective control strategies [8]. Four clusters (Northumberland, Liverpool, Bristol and Leeds) found in this analysis were the subject of investigations undertaken by local outbreak control teams [6,8]. Although not all investigations were published because of the need to protect patient confidentiality, those that were illustrate cluster diversity. The outbreak in a socially deprived area of Northumberland was concerned mainly with young heterosexual adults, predominantly young females [6]. The three year investigation detected 360 cases of gonorrhoea within a locally discrete population. An outbreak of high level azithromycin resistant gonorrhoea emerged amongst young heterosexuals (<20) in Leeds at the end of 2014. Subsequent infections were predominantly seen in MSM aged 18 to 31 residing in London and the South East [37].

PN is a key intervention strategy that is most effective when the time between notification and treatment is minimized [13]. However, the proportion of patients attending clinics as a partner of an index case was highly variable. Specialized sexual health services are invariably located in urban areas. Several clusters seen in this analysis were either outside urban areas or did not border endemic areas [14,38]. Ensuring rural populations have access to care could increase PN rates.

Over the past two decades the epidemiology of gonorrhoea has changed significantly, influenced by population flow, sero-adaptive behaviour among HIV-positive MSM, antimicrobial resistance, advances in diagnostic techniques and therapeutic agents, chemsex, and location-based sexual networking applications [21,39]. As a result, the structure of sexual networks is being transformed from a density-dependent factor into a density-independent factor, thereby increasing the potential for infection transmission. Such developments make control increasingly challenging, particularly in locations where infection is endemic. However, whilst social media and geospatial applications are a constantly evolving forum allowing users to share information from discussing sex and locating sex partners, they are also used to interact with health services, promotion campaigns and testing services. Global mass communication provides an opportunity to explore conversations and factors that influence people’s awareness to sexual health and interaction with clinical services in ways that were not previously possible.

## Conclusions

Heterogeneity across the gonorrhoea epidemic emphasises the importance of local space-time analyses to plan and evaluate sexual health services as a starting point for public health investigation, hypothesis generation, and research. The future challenge is adapting these analytical and visualisation techniques to create an evidence base that enables healthcare professionals to respond to changes within the developing epidemic.

## Supporting information

S1 Text SaTScan parameters for spatial-temporal identification of gonorrhoea clusters. (PDF)

S1 Fig Rate of gonorrhoea diagnoses, England: 2012 & 2013. Regions are separated by local authority (LA). Regions are compared to the English average (100.8 diagnoses per 100,000; 95% CI: 100.0, 101.7) as higher, similar or lower. Similar regions were denoted as those within 20% above or below the English average (100–119 and 80–99 diagnoses per 100,000 people, respectively).
(TIF)

S2 Fig Gonorrhoea diagnosis rate by gender, England: 2012 & 2013. Regions are grouped by LA. Regions are compared to the English average (100.8 diagnoses per 100,000; 95% CI: 100.0, 101.7) as higher, similar or lower. Similar regions were denoted as 20% above or below the English average (100–119 and 80–99 diagnoses per 100,000 people, respectively).
(TIF)

S3 Fig Quarterly gonorrhoea diagnosis rate with regions grouped by middle-layer super output area (MSOA), England: 2013. Regions are compared to the English average (12.6/100,000 people) as higher, similar or lower. Similar regions were denoted as those within 20% above or below the English average (12.6–15.1 and 10.1–12.6/100,000 people, respectively).
(TIF)

S4 Fig Proportion of partner notified gonorrhoea diagnoses by LA contributing to total gonorrhoea diagnoses for males or females. Regions are compared to overall English average proportion of positive partner associated gonorrheoa diagnoses (14.5%, 95% CI: 14.2%, 14.8%) spread evenly across all 326 LAs (0.045% expected per LA). Similar regions were denoted as those within 20% above or below the average per LA (0.036%-0.045% and 0.045%-0.053% partner related diagnoses for a given gender).
(TIF)

S1 Table Identified gonorrhoea endemic regions and clusters with their participating MSOAs, England: 2012 & 2013. Endemic regions were chosen by locations that were participating in a high rate cluster every six months. Outbreak clusters were selected based on a statstical level of *p*<0.05. The cluster numbers correspond to the clusters in Fig 2.
(DOCX)
