# Supplementary material for: Spatial clusters of gonorrhoea in England with particular reference to the outcome of partner notification: 2012 and 2013
Source: PLoS One. 2018 Apr 2;13(4):e0195178. doi: 10.1371/journal.pone.0195178 (PMC5880387; doi:10.1371/journal.pone.0195178)
Supplement: S1 Text — (PDF) [file pone.0195178.s001.pdf]

General Options:

Time precision: Day

Study period: 2012 & 2013

Coordinates: Cartesian

Type of analysis: Retrospective space-time

Time aggregation: 7 days

Probability model: Discrete Poisson

Scan for areas with: High rates

Advanced Analysis Options:

Spatial cluster size: 1% of population at risk

Maximum temporal cluster size: 183 days
