# Supplementary figures and images for: Spatial clusters of gonorrhoea in England with particular reference to the outcome of partner notification: 2012 and 2013
Source: PLoS One. 2018 Apr 2;13(4):e0195178. doi: 10.1371/journal.pone.0195178 (PMC5880387; doi:10.1371/journal.pone.0195178)

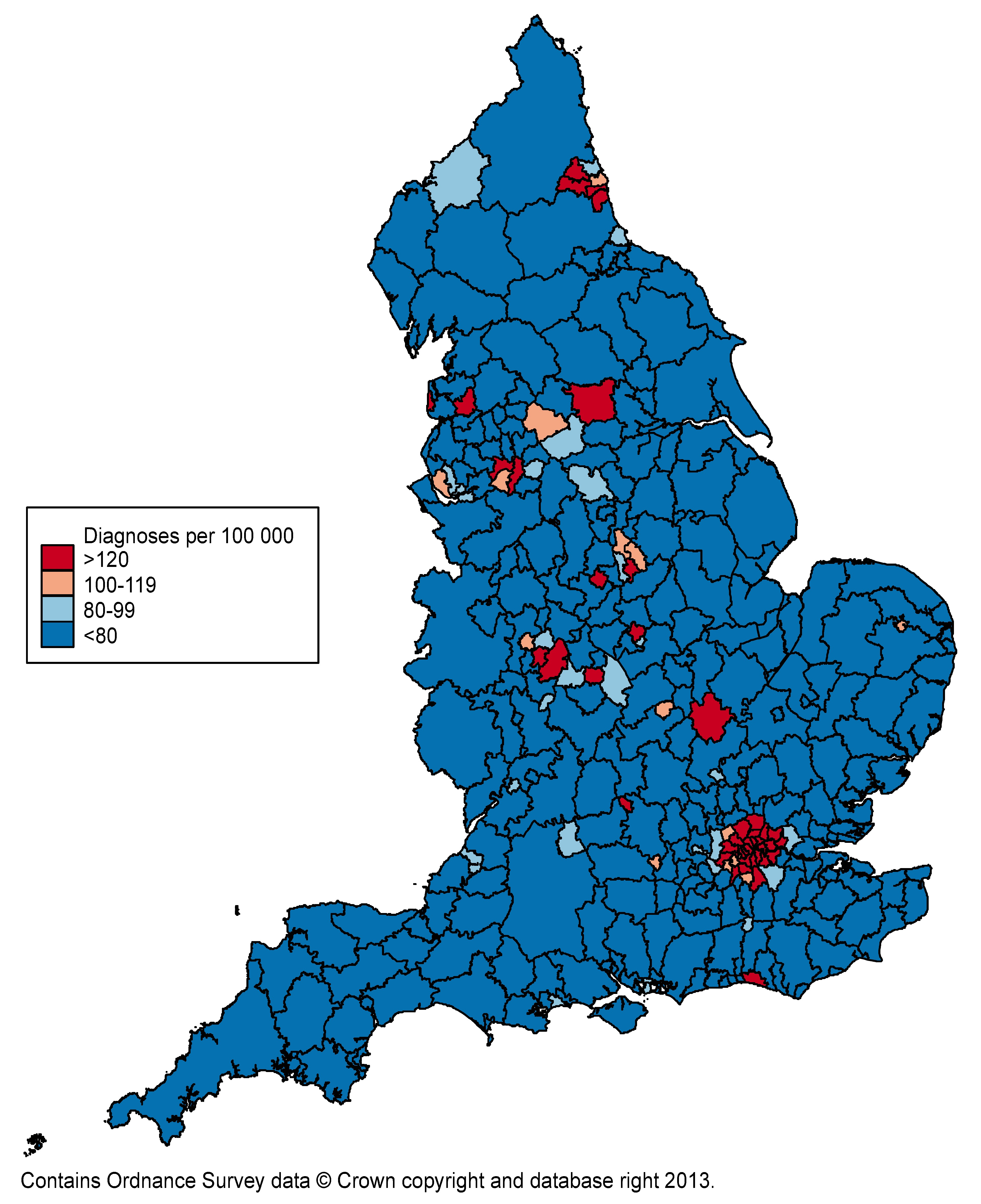

Supplement: S1 Fig — Regions are separated by local authority (LA). Regions are compared to the English average (100.8 diagnoses per 100,000; 95% CI: 100.0, 101.7) as higher, similar or lower. Similar regions were denoted as those within 20% above or below the English average (100–119 and 80–99 diagnoses per 100,000 people, respectively). (TIF) [file pone.0195178.s002.tif]

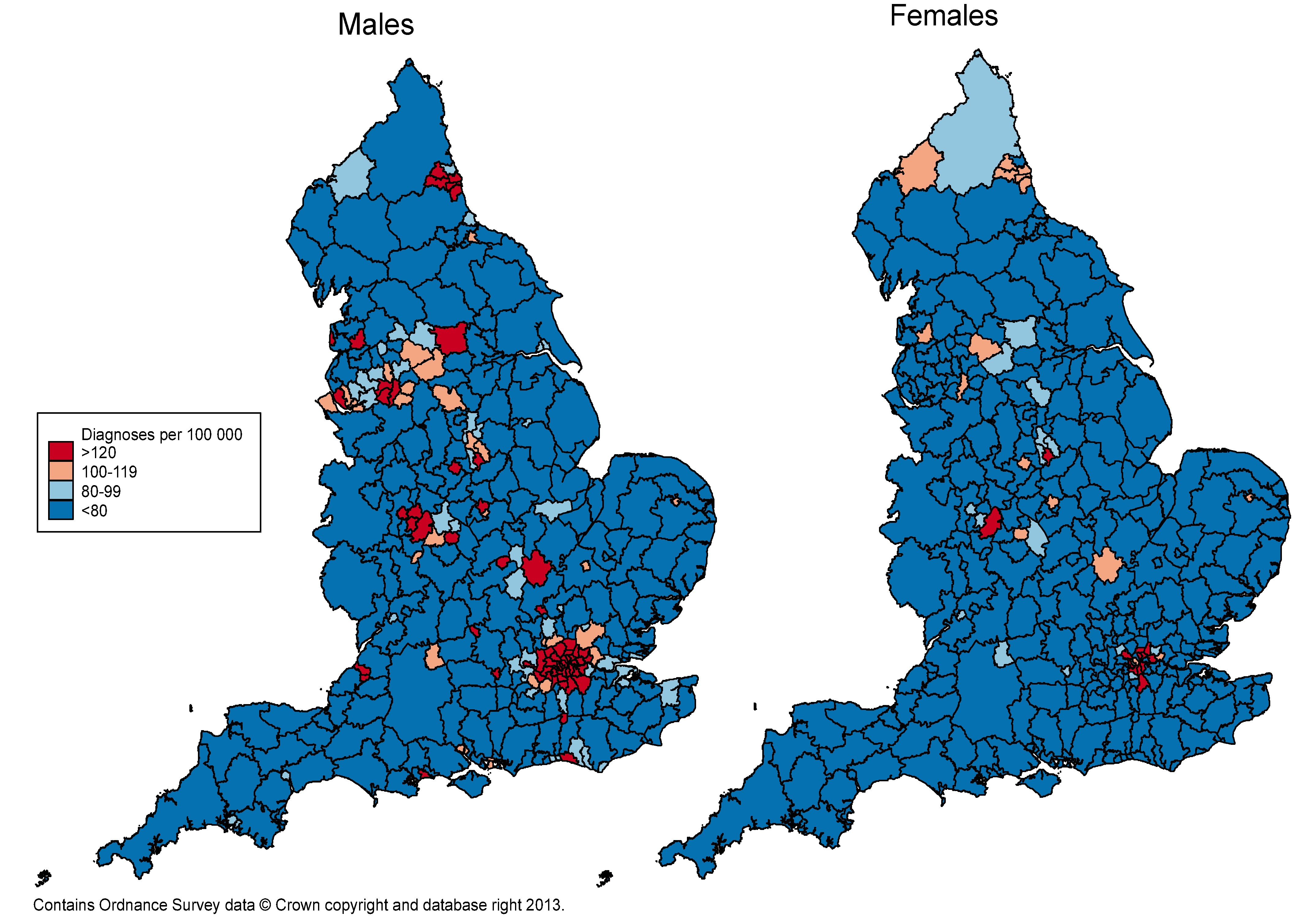

Supplement: S2 Fig — Regions are grouped by LA. Regions are compared to the English average (100.8 diagnoses per 100,000; 95% CI: 100.0, 101.7) as higher, similar or lower. Similar regions were denoted as 20% above or below the English average (100–119 and 80–99 diagnoses per 100,000 people, respectively). (TIF) [file pone.0195178.s003.tif]

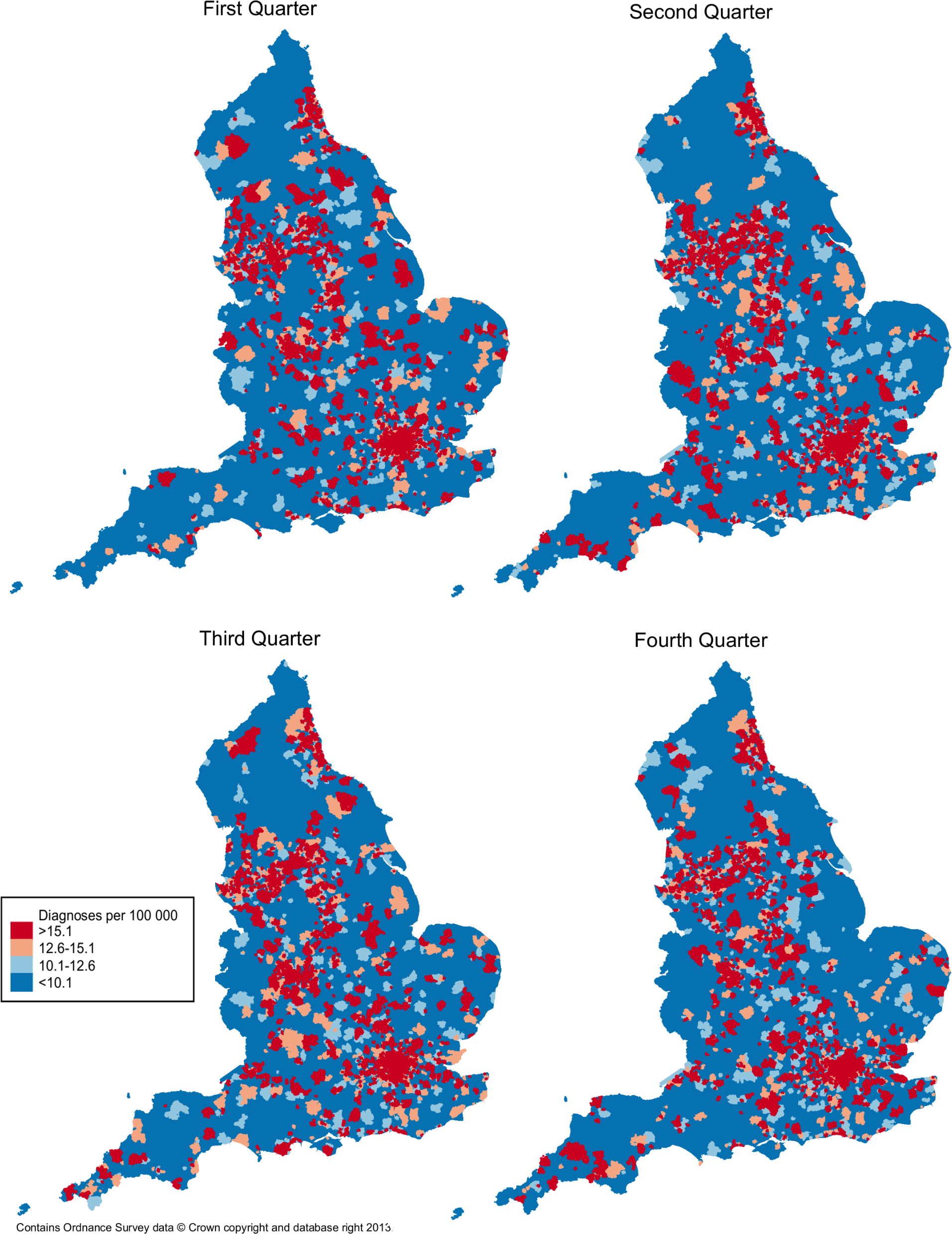

Supplement: S3 Fig — Regions are compared to the English average (12.6/100,000 people) as higher, similar or lower. Similar regions were denoted as those within 20% above or below the English average (12.6–15.1 and 10.1–12.6/100,000 people, respectively). (TIF) [file pone.0195178.s004.tif]

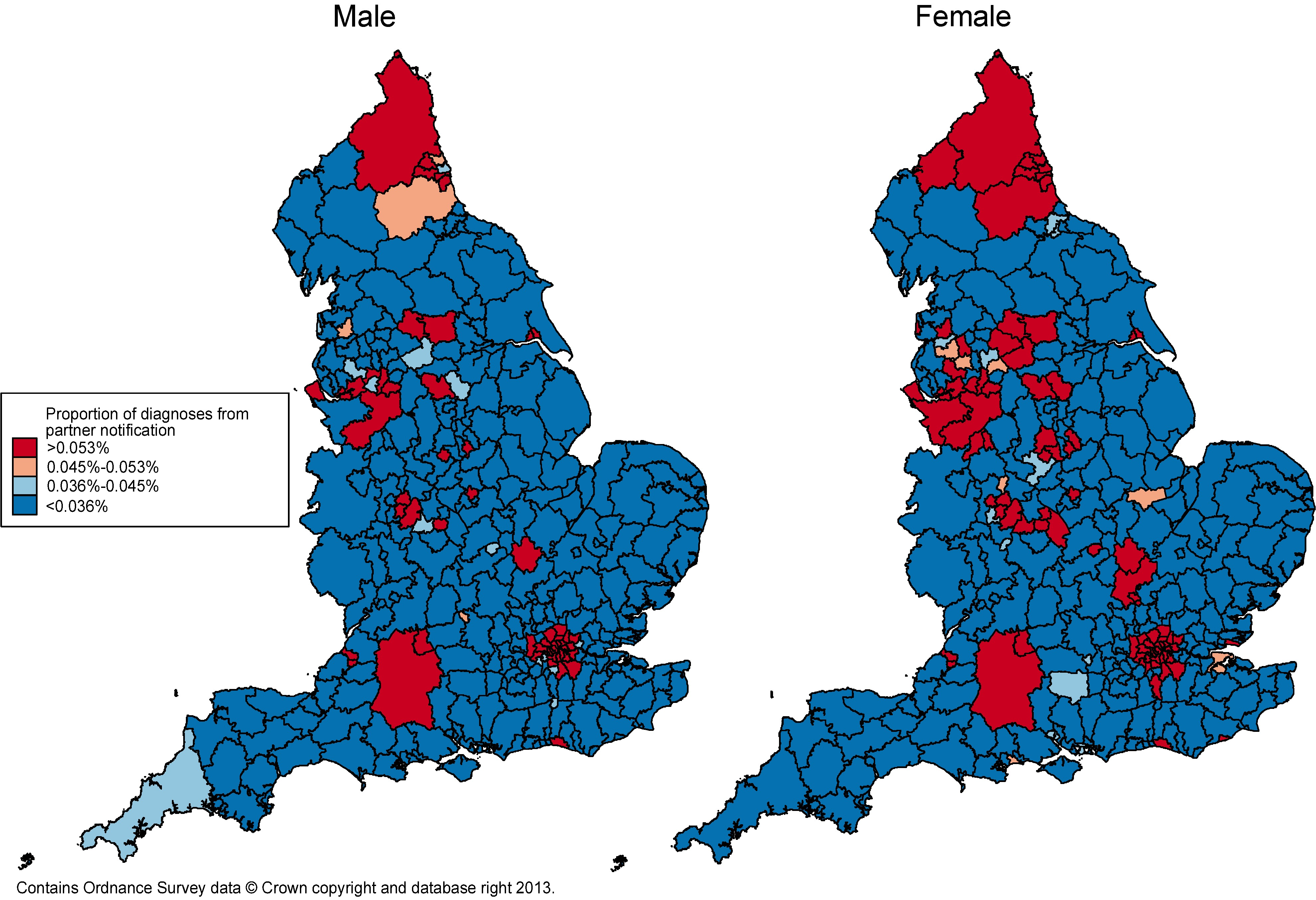

Supplement: S4 Fig — Regions are compared to overall English average proportion of positive partner associated gonorrheoa diagnoses (14.5%, 95% CI: 14.2%, 14.8%) spread evenly across all 326 LAs (0.045% expected per LA). Similar regions were denoted as those within 20% above or below the average per LA (0.036%-0.045% and 0.045%-0.053% partner related diagnoses for a given gender). (TIF) [file pone.0195178.s005.tif]
