# Supplementary material for: Spatial clusters of gonorrhoea in England with particular reference to the outcome of partner notification: 2012 and 2013
Source: PLoS One. 2018 Apr 2;13(4):e0195178. doi: 10.1371/journal.pone.0195178 (PMC5880387; doi:10.1371/journal.pone.0195178)
Supplement: S1 Table — Endemic regions were chosen by locations that were participating in a high rate cluster every six months. Outbreak clusters were selected based on a statstical level of p<0.05. The cluster numbers correspond to the clusters in Fig 2. (DOCX) [file pone.0195178.s006.docx]

| **Regions** | | **Participating MSOA region(s)** | **RR^*^ (p-value)** |
| --- | --- | --- | --- |
| **Endemic Regions** | 1 | Barking and Dagenham, Barnet, Brent, Camden, Hackney, Hammersmith and Fulham, Haringey, Islington, Kensington and Chelsea, Lambeth, Newham, Redbridge, Southwark, Tower Hamlets, Wandsworth, Westminster | - |
|  | 2 | Manchester, Trafford, Salford | - |
|  | 3 | Birmingham | - |
|  | 4 | Brighton and Hove | - |
| **Cluster Regions** | 1 | Camden, City of London, Hackney, Islington, Lambeth, Southwark, Tower Hamlets, Waltham Forest | 5.7 (<0.0001) |
|  | 2 | Hammersmith and Fulham, Kensington and Chelsea, Lambeth, Wandsworth, Westminster | 4.5 (<0.0001) |
|  | 3 | Barnet, Brent, Camden, Ealing, Hammersmith and Fulham, Kensington and Chelsea, Westminster | 3.7 (<0.0001) |
|  | 4 | Brighton and Hove | 4.9 (<0.0001) |
|  | 5 | Bexley, Greenwich, Lewisham, Newham, Tower Hamlets | 3.3 (<0.0001) |
|  | 6 | Ashfield, Gedling, Nottingham | 3.1 (<0.0001) |
|  | 7 | Bromley, Croydon, Lambeth, Merton | 2.8 (<0.0001) |
|  | 8 | Manchester, Oldham, Salford, Stockport, Tameside, Trafford | 2.8 (<0.0001) |
|  | 9 | Birmingham, Sandwell, Solihull | 2.7 (<0.0001) |
|  | 10 | County Durham, Gateshead, Newcastle upon Tyne, North Tyneside, South Tyneside, Sunderland | 2.6 (<0.0001) |
|  | 11 | Leeds | 2.6 (<0.0001) |
|  | 12 | Bradford, Calderdale, Kirklees | 2.8 (<0.0001) |
|  | 13 | Ealing, Hounslow, Richmond upon Thames | 2.8 (<0.0001) |
|  | 14 | Enfield, Haringey, Waltham Forest | 2.3 (<0.0001) |
|  | 15 | Rotherham, Sheffield | 2.7 (<0.0001) |
|  | 16 | Coventry | 2.8 (<0.0001) |
|  | 17 | Dudley, Sandwell, South Staffordshire, Walsall, Wolverhampton | 2.0 (<0.0001) |
|  | 18 | Derby | 4.2 (<0.0001) |
|  | 19 | Kingston upon Thames, Merton, Sutton, Wandsworth | 2.0 (<0.0001) |
|  | 20 | Blackpool, Wyre | 3.4 (<0.0001) |
|  | 21 | Barking and Dagenham, Epping Forest, Newham, Redbridge, Waltham Forest | 1.9 (<0.0001) |
|  | 22 | Leicester | 2.3 (<0.0001) |
|  | 23 | Liverpool | 2.8 (<0.0001) |
|  | 24 | Birmingham, Bromsgrove, Dudley | 2.3 (<0.0001) |
|  | 25 | Nottingham | 9.6 (<0.0001) |
|  | 26 | Northumberland | 3.1 (<0.0001) |
|  | 27 | Bolton, Bury, Salford, Trafford, Warrington, Wigan | 1.8 (0.001) |
|  | 28 | Preston | 3.5 (0.002) |
|  | 29 | Barnet, Brent, Harrow | 2.3 (0.002) |
|  | 30 | Bradford, Leeds | 3.0 (0.002) |
|  | 31 | Bedford | 4.8 (0.006) |
|  | 32 | Hillingdon, Hounslow, Slough, Spelthorne | 2.6 (0.02) |
|  | 33 | Ashfield, Bolsover, Mansfield | 3.1 (0.02) |

*RR = risk ratio
